# Recurrent disease detection after resection of pancreatic ductal adenocarcinoma using a recurrence-focused surveillance strategy (RADAR-PANC): protocol of an international randomized controlled trial according to the Trials within Cohorts design

**DOI:** 10.1186/s13063-024-08223-5

**Published:** 2024-06-20

**Authors:** L. A. Daamen, I. W. J. M. van Goor, V. P. Groot, P. C. M. Andel, L. A. A. Brosens, O. R. Busch, G. A. Cirkel, N. Haj Mohammad, H. D. Heerkens, I. H. J. T. de Hingh, F. Hoogwater, H. W. M. van Laarhoven, M. Los, G. J. Meijer, V. E. de Meijer, R. Pande, K. J. Roberts, J. Stoker, M. W. J. Stommel, G. van Tienhoven, R. C. Verdonk, H. M. Verkooijen, F. J. Wessels, J. W. Wilmink, M. G. Besselink, H. C. van Santvoort, M. P. W. Intven, I. Q. Molenaar, for the Dutch Pancreatic Cancer Group

**Affiliations:** 1 Department of Surgery, Regional Academic Cancer Center Utrecht, UMC Utrecht Cancer Center & St. Antonius Hospital Nieuwegein, Nieuwegein, the Netherlands; 2 Division of Imaging, UMC Utrecht Cancer Center, Utrecht University, Utrecht, the Netherlands; 3 Department of Radiation Oncology, Regional Academic Cancer Center Utrecht, UMC Utrecht Cancer Center & St. Antonius Hospital Nieuwegein, Nieuwegein, the Netherlands; 4 Department of Pathology, Regional Academic Cancer Center Utrecht, UMC Utrecht Cancer Center & St. Antonius Hospital Nieuwegein, Nieuwegein, the Netherlands; 5 Department of Pathology, Radboud University Medical Center, Nijmegen, the Netherlands; 6 Amsterdam UMC, Department of Surgery, Location University of Amsterdam, Amsterdam, the Netherlands; 7 Cancer Center Amsterdam, Amsterdam, the Netherlands; 8 Department of Medical Oncology, Regional Academic Cancer Center Utrecht, UMC Utrecht Cancer Center, St. Antonius Hospital Nieuwegein & Meander Medical Center, Utrecht University, Utrecht, the Netherlands; 9 Department of Radiation Oncology, Radboud University Medical Center, Nijmegen, the Netherlands; 10 Department of Surgery, Catharina Hospital, Eindhoven, the Netherlands; 11 Department of Surgery, University of Groningen, University Medical Center Groningen, Groningen, the Netherlands; 12 Amsterdam UMC, Department of Medical Oncology, Location University of Amsterdam, Amsterdam, the Netherlands; 13 Department of Hepatopancreatobiliary Surgery and Liver Transplantation, Queen Elizabeth Hospital, University Hospitals Birmingham, Birmingham, UK; 14 Institute of Immunology and Immunotherapy, University of Birmingham, Birmingham, UK; 15 Amsterdam UMC, Department of Radiology, Location University of Amsterdam, Amsterdam, the Netherlands; 16 Department of Surgery, Radboud University Medical Center, Nijmegen, the Netherlands; 17 Amsterdam UMC, Department of Radiation Oncology, Location University of Amsterdam, Amsterdam, the Netherlands; 18 Department of Gastroenterology and Hepatology, Regional Academic Cancer Center Utrecht, UMC Utrecht Cancer Center & St. Antonius Hospital Nieuwegein, Nieuwegein, the Netherlands; 19 Department of Radiology, Regional Academic Cancer Center Utrecht, UMC Utrecht Cancer Center & St. Antonius Hospital Nieuwegein, Nieuwegein, the Netherlands

**Keywords:** Pancreatic cancer, Pancreatic ductal adenocarcinoma, PDAC, Postoperative surveillance, Disease recurrence, Survival, Quality of life

## Abstract

**Background:**

Disease recurrence remains one of the biggest concerns in patients after resection of pancreatic ductal adenocarcinoma (PDAC). Despite (neo)adjuvant systemic therapy, most patients experience local and/or distant PDAC recurrence within 2 years. High-level evidence regarding the benefits of recurrence-focused surveillance after PDAC resection is missing, and the impact of early detection and treatment of recurrence on survival and quality of life is unknown. In most European countries, recurrence-focused follow-up after surgery for PDAC is currently lacking. Consequently, guidelines regarding postoperative surveillance are based on expert opinion and other low-level evidence. The recent emergence of more potent local and systemic treatment options for PDAC recurrence has increased interest in early diagnosis. To determine whether early detection and treatment of recurrence can lead to improved survival and quality of life, we designed an international randomized trial.

**Methods:**

This randomized controlled trial is nested within an existing prospective cohort in pancreatic cancer centers in the Netherlands (Dutch Pancreatic Cancer Project; PACAP) and the United Kingdom (UK) (Pancreas Cancer: Observations of Practice and survival; PACOPS) according to the “Trials within Cohorts” (TwiCs) design. All PACAP/PACOPS participants with a macroscopically radical resection (R0-R1) of histologically confirmed PDAC, who provided informed consent for TwiCs and participation in quality of life questionnaires, are included. Participants randomized to the intervention arm are offered recurrence-focused surveillance, existing of clinical evaluation, serum cancer antigen (CA) 19–9 testing, and contrast-enhanced computed tomography (CT) of chest and abdomen every three months during the first 2 years after surgery. Participants in the control arm of the study will undergo non-standardized clinical follow-up, generally consisting of clinical follow-up with imaging and serum tumor marker testing only in case of onset of symptoms, according to local practice in the participating hospital. The primary endpoint is overall survival. Secondary endpoints include quality of life, patterns of recurrence, compliance to and costs of recurrence-focused follow-up, and the impact on recurrence-focused treatment.

**Discussion:**

The RADAR-PANC trial will be the first randomized controlled trial to generate high level evidence for the current clinical equipoise regarding the value of recurrence-focused postoperative surveillance with serial tumor marker testing and routine imaging in patients after PDAC resection. The Trials within Cohort design allows us to study the acceptability of recurrence-focused surveillance among cohort participants and increases the generalizability of findings to the general population. While it is strongly encouraged to offer all trial participants treatment at time of recurrence diagnosis, type and timing of treatment will be determined through shared decision-making. This might reduce the potential survival benefits of recurrence-focused surveillance, although insights into the impact on patients’ quality of life will be obtained.

**Trial registration:**

Clinicaltrials.gov, NCT04875325. Registered on May 6, 2021.

## Administrative information

Note: the numbers in curly brackets in this protocol refer to SPIRIT checklist item numbers. The order of the items has been modified to group similar items (see http://www.equator-network.org/reporting-guidelines/spirit-2013-statement-defining-standard-protocol-items-for-clinical-trials/).

**Table:** 

Title {1}	Recurrent diseAse Detection After Resection of PANCreatic ductal adenocarcinoma using a recurrence-focused surveillance strategy (RADAR-PANC) – protocol of an international randomized controlled trial according to the Trials within Cohorts design
Trial registration {2a and 2b}.	Clinicaltrials.gov registration, NCT04875325. Registered on 6 May 2021, https://clinicaltrials.gov/study/NCT04875325?term=NCT04875325&rank=1
Protocol version {3}	Version 4.0, October 24, 2023
Funding {4}	Funded by Deltaplan Alvleesklierkanker
Author details {5a}	1. Department of Surgery, Regional Academic Cancer Center Utrecht, UMC Utrecht Cancer Center & St. Antonius Hospital Nieuwegein, Nieuwegein, The Netherlands. 2. Division of Imaging, UMC Utrecht Cancer Center, Utrecht University, Utrecht, The Netherlands. 3. Department of Radiation Oncology, Regional Academic Cancer Center Utrecht, UMC Utrecht Cancer Center & St. Antonius Hospital Nieuwegein, Nieuwegein, The Netherlands. 4. Department of Pathology, Regional Academic Cancer Center Utrecht, UMC Utrecht Cancer Center & St. Antonius Hospital Nieuwegein, Nieuwegein, The Netherlands. 5. Department of Pathology, Radboud University Medical Center, Nijmegen, the Netherlands. 6. Amsterdam UMC, location University of Amsterdam, department of surgery, Amsterdam, the Netherlands. 7. Cancer Center Amsterdam, the Netherlands. 8. Department of Medical Oncology, Regional Academic Cancer Center Utrecht, UMC Utrecht Cancer Center, St. Antonius Hospital Nieuwegein & Meander Medical Center, Utrecht University, the Netherlands. 9. Department of Radiation Oncology, Radboud University Medical Center, Nijmegen, the Netherlands. 10. Department of Surgery, Catharina Hospital, Eindhoven, the Netherlands. 11. Department of Surgery, University of Groningen, University Medical Center Groningen, Groningen, the Netherlands. 12. Amsterdam UMC, location University of Amsterdam, department of medical oncology, Amsterdam, the Netherlands. 13. Department of Hepatopancreatobiliary Surgery and Liver Transplantation, Queen Elizabeth Hospital, University Hospitals Birmingham, Birmingham, United Kingdom. 14. Department of Hepatopancreatobiliary Surgery and Liver Transplantation, Queen Elizabeth Hospital, University Hospitals Birmingham, Birmingham, United Kingdom; Institute of Immunology and Immunotherapy, University of Birmingham, Birmingham, United Kingdom. 15. Amsterdam UMC, location University of Amsterdam, department of radiology, Amsterdam, the Netherlands. 16. Department of Surgery, Radboud University Medical Center, Nijmegen, the Netherlands. 17. Amsterdam UMC, location University of Amsterdam, department of radiation oncology, Amsterdam, the Netherlands. 18. Department of Gastroenterology and Hepatology, Regional Academic Cancer Center Utrecht, UMC Utrecht Cancer Center & St. Antonius Hospital Nieuwegein, Nieuwegein, The Netherlands. 19. Department of Radiology, Regional Academic Cancer Center Utrecht, UMC Utrecht Cancer Center & St. Antonius Hospital Nieuwegein, Nieuwegein, The Netherlands.
Name and contact information for the trial sponsor {5b}	UMC Utrecht PO Box 85,500 3508 GA Utrecht, The Netherlands
Role of sponsor {5c}	Overseeing and coordinating the clinical trial, facilitating funding sources and collecting and analyzing the data.

## Introduction

### Background and rationale {6a}

#### Background

Pancreatic ductal adenocarcinoma (PDAC) is the fourth leading cause of cancer related mortality in Europe for both men and women and is expected to rise to the second position by 2030 [1, 2]. For patients with localized disease, radical resection combined with systemic therapy offers the best chance of long-term survival and can be curative in some cases [3, 4]. However, almost all patients experience local and/or distant disease recurrence at some point during follow-up [5–8]. Therefore, even after PDAC resection, median overall survival remains less than 2 years, with only 4% of patients surviving over 10 years [3, 9].

Current research is mainly focused on management of primary or metastatic PDAC, with most trials evaluating treatment strategies for primary resectable or locally advanced PDAC. Studies regarding the efficacy and optimal timing of treatment for recurrence are scarce. As a result, prospective evidence on survival benefits following early detection and treatment of recurrence is lacking and the effect on quality of life (QoL) remains unknown. This has led to conflicting recommendations on follow-up after resection of PDAC, and current PDAC guidelines regarding postoperative surveillance are based on expert opinion and other low-level evidence (Table 1) [10–16].

**Table 1:** Current guidelines and follow-up

Medical society	Recommendation	Level of evidence
National Comprehensive Cancer Network (NCCN—2018) [11]	Clinical evaluation every 3–6 months for 2 years, then annually	Uniform expert opinion (2A)
	CA 19–9, CT scan every 3–6 months for 2 years, then annually	Non-uniform expert opinion (2B)
European Society for Medical Oncology (ESMO—2015) [12]	No evidence that regular follow-up after therapy with curative intent has any impact on outcome	No evidence (4D)
	Follow-up should concentrate on symptoms, nutrition, and psycho-social support	No evidence (4D)
Japan Pancreas Society (2013) [13]	Measurement of tumor markers and a dynamic CT scan every 3 to 6 months for 2 years postoperatively and every 6 to 12 months subsequently, at least for 5 years postoperatively	Low (C)
American Society of Clinical Oncology (ASCO—2017) [14]	History and physical evaluation every 3–6 months after completion of therapy. Additional serum CA 19–9 if elevated pre-operatively	Uniform expert opinion (2A)
	Benefit of imaging is unclear; it seems to result in greater detection of asymptomatic recurrence	
	Primary focus on treatment-related toxicity, survivorship issues, and recurrence monitoring	
National Dutch Guideline Pancreatic carcinoma (NVvH—2019) [15]	Clinical follow-up. In case of symptoms of disease recurrence, additional diagnostic testing can be used	Uniform expert opinion (2A)
	Consider routine diagnostic follow-up in an investigative context only	Low (C)
International Association of Pancreatology/European Pancreatic Club (IAP/EPC—2016) [16]	No recommendation	Not applicable

Recent introduction of more potent systemic therapies, such as fluorouracil, leucovorin, irinotecan and oxaliplatin (FOLFIRINOX) and gemcitabine/nab-paclitaxel combination therapy, and local ablative therapies, such as stereotactic body radiation therapy (SBRT), has opened the field for treatment of PDAC recurrence [4, 17]. Despite a lack of evidence, this has resulted in an increased interest in recurrence-focused postoperative surveillance with routine imaging and serial serum tumor marker testing, with the ultimate goal to detect PDAC recurrence at an early asymptomatic stage [18]. This hypothetically leads to the identification of patients with a good performance status who are most likely to tolerate (experimental) treatment [19]. However, it remains unclear whether all patients with asymptomatic PDAC recurrence can be treated and whether early initiation of recurrence-focused treatment results in improved oncological outcomes. Moreover, early initiation of recurrence treatment in asymptomatic patients might induce treatment-related toxicity, which could worsen quality of life. Recurrence-focused surveillance could also increase fear of cancer recurrence, which negatively impacts quality of life, as might detection of recurrence in an asymptomatic stage [20, 21]. Consequently, in most European countries including the Netherlands and the United Kingdom (UK), ethical concerns towards a recurrence-focused follow-up are raised and guidelines do not recommend a recurrence-focused follow-up strategy [11, 14].

#### Rationale

The RADAR-PANC trial is designed to investigate whether a recurrence-focused surveillance strategy with serial tumor marker testing and routine imaging improves overall survival in patients after radical resection for PDAC in the Netherlands and the UK, compared to current non-standardized follow-up. Furthermore, the consequences of a recurrence-focused surveillance strategy on quality of life and additional (experimental) treatment will be assessed.

### Objectives {7}

The main objective of this study is to investigate whether recurrence-focused surveillance with three-monthly serum tumor marker testing and computed tomography (CT) imaging improves survival of patients with primary resected PDAC.

We hypothesize that patients who receive recurrence-focused surveillance will be diagnosed with PDAC recurrence at an earlier stage (i.e., localized disease and a good performance status). This hypothetically increases the number of patients that could tolerate (experimental) treatment for recurrence, with subsequent potential survival benefits. The goal of recurrence-focused surveillance will therefore be to improve overall survival.

The secondary objectives of this study are as follows:

- To evaluate the impact of recurrence-focused surveillance on quality of life
- To evaluate compliance to a recurrence-focused surveillance strategy
- To evaluate the impact of recurrence-focused follow-up on worry of cancer recurrence
- To evaluate disease-free survival
- To assess clinical and radiological patterns of PDAC recurrence
- To assess the role of serum tumor marker testing in detecting recurrent PDAC
- To assess eligibility for recurrence-focused (experimental) systemic and/or local treatment
- To evaluate reasons to refrain from treatment for recurrence (i.e., eligibility, deteriorated condition, patient’s wish, doctors’ advice, etc.)
- To assess patients’ tolerance for treatment for recurrence
- To assess prognostic factors for asymptomatic PDAC recurrence
- To calculate the cost-efficacy of a recurrence-focused follow-up

### Trial design {8}

The RADAR-PANC trial is an international randomized controlled trial nested within two large nationwide prospective cohorts (the Dutch Pancreatic Cancer Project; PACAP, and the UK Pancreas Cancer: Observations of Practice and survival; PACOPS) and follows the “Trials within Cohorts” (TwiCs) design [22]. With this design, a large observational cohort of patients is recruited and used as a multiple trials facility, and “patient-centered” information and consent are provided an obtained. For each randomized controlled trial, eligible patients are identified within the cohort and randomized [23]. The TwiCs design improves trial participation by avoiding non-participation on the basis of patients’ or doctors’ preference for the intervention.

In 2013, PACAP was initiated by the Dutch Pancreatic Cancer Group (DPCG) [24]. The UK PACOPS project was established in 2023 and follows the same setup as PACAP. Within these projects, patients with PDAC are asked to participate in the PACAP/PACOPS cohort at diagnosis. First, patients are asked general informed consent for collection of specific data on demographics and clinical, laboratory and radiological findings before, during, and after treatment and follow-up. Second, patients are asked to provide informed consent for participation in quality of life questionnaires to assess Patient Reported Outcome Measurements (PROMs). Third, patients are asked to provide informed consent for being randomized in future studies according to the TwiCs design. At the time informed consent is signed, neither the patient nor the researcher knows for which studies this patient is or might become eligible. This is called a “broad” consent, since patients do not know for which specific research questions their data may be used in the future [23]. Participants are only informed in case they can actually receive the intervention, i.e., when randomized to the intervention arm. If they then choose to undergo the intervention, they provide additional informed consent (two-staged informed consent) [25]. Patients randomized to the control arm are not further notified and followed in the context of the prospective cohort study, according to the current, non-standardized best practice. This approach is thought to reduce disappointment bias and cross-over of patients randomized to the control arm of the study. Importantly, at cohort enrolment, patients are adequately informed that at some point during their treatment or follow-up, they may be serving as control without being explicitly notified, which was demonstrated to be positively or neutrally received among patients [26].

## Methods: participants, interventions, and outcomes

### Study setting {9}

International, multicenter randomized controlled trial (NCT04875325).

### Eligibility criteria {10}

Patients are eligible for randomization if they meet all of the following criteria:

1. Participation in the PACAP or PACOPS cohort
2. Macroscopic radical resection (R0-R1) of histologically confirmed PDAC
3. Age ≥ 18 years
4. Written informed consent for being randomized in future studies (TwiCs) and participation in quality of life questionnaires (PROMs)

A potential study participant who meets any of the following criteria is excluded:

- Contraindications for contrast-enhanced CT imaging, following the protocol of the department of radiology in each participating hospital
- Mentally or physically incapable of giving consent
- Participation in other studies with a study-specific follow-up comprising imaging and/or serum tumor marker testing

### Who will take informed consent? {26a}

**Fig. 1:**
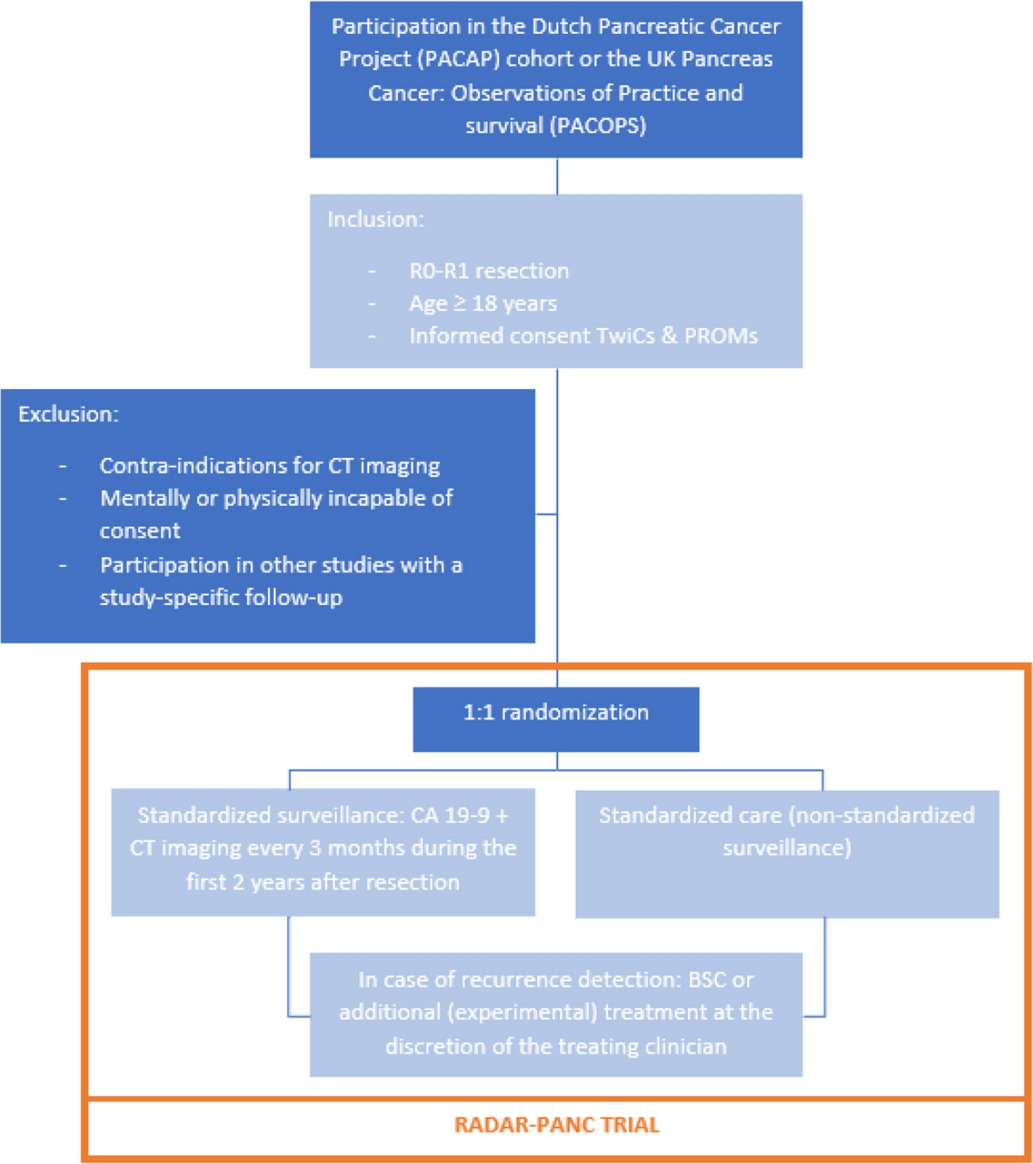
Schematic inclusion and randomization process of the RADAR-PANC trial. PDAC pancreatic ductal adenocarcinoma, TwiCs Trials within Cohorts, PROMs patient-reported outcome measures, CT computed tomography, CA carbohydrate antigen, BSC best supportive care

The inclusion and randomization process of the RADAR-PANC trial is schematically shown in Fig. 1. Potentially eligible subjects are identified by the (local) investigator or an authorized delegate, by checking pathology reports of patients with known or suspected PDAC after intended radical resection. The (local) investigator or an authorized delegate will confirm that a potentially eligible patient is included in the PACAP or PACOPS cohort and signed informed consent for being randomized in future studies according to the TwiCs design and participation in quality of life questionnaires. If not, the patient will continue standard of care and will not be randomized for this trial. If the patient meets all the eligibility criteria for the RADAR-PANC trial, the participant is randomized for postoperative surveillance strategy with a 1:1 allocation ratio. Participants are either randomized for follow-up according to the current, non-standardized practice (control), or recurrence-focused surveillance according to the trial protocol (intervention). If the patient is randomized to the control arm, no additional action is taken: patients will receive current standard of care and are not explicitly informed about being randomized to the control arm of the trial according to the TwiCs design. Patients randomized to the intervention arm of the trial will be approached by a researcher and will be offered the experimental intervention within the first 3 to 4 months post-surgery. These patients are informed that they are free to decide whether they want to accept or deny the intervention. Oral and written information is provided and the treating physician is informed. If the patient chooses to participate, the additional informed consent form is signed prior to the start of the intervention.

### Additional consent provisions for collection and use of participant data and biological specimens {26b}

All participants in the RADAR-PANC trial are already participating in PACAP or PACOPS and have already provided broad informed consent for the use of their medical data prior to inclusion in the current trial. Consequently, no additional consent for further studies is needed for collection and use of participant data.

### Interventions

#### Explanation for the choice of comparators {6b}

Participants in the control arm of the RADAR-PANC trial will receive non-standardized clinical follow-up, according to local practice in the participating hospital. As the most recent version of the national Dutch guideline for PDAC care currently does not recommend a recurrence-focused follow-up frequency, methodology, or focus, follow-up after PDAC resection is based on shared decision-making, at the discretion of the treating clinician and the patients’ wish [15].

### Intervention description {11a}

The intervention exists of a recurrence-focused follow-up approach with routine imaging and serial serum tumor marker testing. The follow-up scheme of the RADAR-PANC trial is based on current surveillance guidelines after resection for PDAC as recommended by the National Comprehensive Cancer Network (NCCN) [11]. This intense follow-up scheme was chosen to increase the chance of early diagnosis of disease recurrence. For the first 2 years after surgery, clinical evaluation, serum carbohydrate antigen 19–9 (CA 19–9) testing, and contrast-enhanced CT imaging of chest and abdomen is performed every 3 months.

Follow-up of patients and related procedures (i.e., blood draws, CT scans) in this study can be performed at the participating hospital, referring hospital, or central coordinating hospital. Study-related follow-up is performed at 3, 6, 9, 12, 15, 18, 21, and 24 months after surgery. For the first follow-up evaluation, patients need to be randomized within the first 3 to 4 months post-surgery. Follow-up appointments are made by the trial coordinator in consultation with the treating clinician. If possible, outpatient clinic visits are combined with CT scan appointments; blood samples are obtained simultaneously.

#### Blood samples

At 3 months postoperatively and every subsequent 3 months during the first 2 years of follow-up, blood samples are obtained from study participants to assess CA 19–9 values. In addition, serum bilirubin is also determined to assess adjusted CA 19–9 levels.

#### CT imaging

During the first 2 years of follow-up, a CT of chest and abdomen with intravenous contrast (late arterial phase of the upper abdomen and portal venous phase covering the entire abdomen) is performed at 3 months postoperatively and every subsequent 3 months.

#### Quality of life assessment

All study participants, either randomized for the intervention or control arm of the RADAR-PANC trial, will receive quality of life questionnaires at each follow-up appointment, in the context of the PACAP and PACOPS cohort studies. The following questionnaires are used: non-disease specific health-related quality of life (HRQL) (EQ-5D-5L), cancer-specific HRQL (EORTC QLQ-C30), tumor-specific HRQL (EORTC QLQ-PAN26), hospital anxiety and depression scale (HADS), and worry of progression of cancer scale (WOPS; modified Dutch seven-item version of the six-item English cancer worry scale (CWS)) [27–31].

#### Recurrence diagnosis

If disease recurrence (local and/or distant) is suspected on the basis of a follow-up CT scan, with or without elevated tumor markers, pathological confirmation is strongly advised. If the results remain inconclusive or pathology cannot be obtained, additional imaging (magnetic resonance imaging (MRI) and/or positron emission tomography CT (PET-CT)) may be considered. Consensus on the presence of a recurrence should be obtained in a multidisciplinary meeting (e.g., based on imaging, elevated CA 19–9, and clinical evaluation).

If disease recurrence is diagnosed, best supportive care (BSC) or systemic and/or local therapy is offered at the discretion of the treating clinician in both trial arms, which is the current standard practice, and trial-related follow-up will end. It is encouraged to offer treatment to all patients in whom there are no contraindications based on clinical condition (performance status), comorbidity, or specific exclusion criteria for systemic and/or local therapy. Patients can be offered to participate in intervention trials on management of pancreatic cancer recurrence. In the Netherlands, within the DPCG, two intervention studies for the treatment of PDAC recurrence have been simultaneously initiated: the ARCADE trial (NCT04881487), investigating the efficacy of stereotactic body radiation therapy on survival after recurrence and quality of life in patients with isolated local PDAC recurrence, and the TIMEPAN trial (NCT04897854) studying the optimal timing of start of systemic treatment for asymptomatic metastasized PDAC.

### Criteria for discontinuing or modifying allocated interventions {11b}

Participants may withdraw from participation in the RADAR-PANC study at any time and for any reason without consequence. The principal investigator may withdraw a patient from the study for one or more of the following reasons: the patient is not following the research staff's instructions of the protocol, continued participation could be harmful to the patient, and other administrative reasons or unforeseen circumstances.

If participants become too ill to attend their follow-up visits, the study team will continue to send quality of life questionnaires and obtain data through the patients’ general practitioner.

### Strategies to improve adherence to interventions {11c}

For full adherence to the study protocol, the trial coordinator is responsible for arranging all follow-up appointments and diagnostic tests for patients, in consultation with the treating physician at the participating or referring center. Diagnostic testing can be performed either at the local participating center as well as at the referring center or the central coordinating center, as desired by the patient. The trial coordinator will monitor whether follow-up measurements have been completed, including diagnostic tests as well as quality of life questionnaires.

### Relevant concomitant care permitted or prohibited during the trial {11d}

Concomitant care and interventions will not be prohibited during the trial.

### Provisions for post-trial care {30}

Individual patients will remain enrolled in the RADAR-PANC trial for a maximum of 2 years or until patient withdrawal. Follow-up of patients that have completed the 2-year follow-up will be continued for survival information until death. Patients who are withdrawn from the study will be followed-up by a medical specialist or general practitioner for survival information until death. After the study period has ended for an individual patient, continuation of either recurrence-focused or non-standardized follow-up and diagnostic testing is based on shared decision-making between patients and their treating physicians.

### Outcomes {12}

The primary outcome is overall survival, defined as the time from the date of PDAC resection until the date of either death from any cause or last follow-up.

Secondary outcomes are the following:

- Patient-reported quality of life and worry of cancer recurrence, as measured by PROMs questionnaires within the PACAP-PROMs and PACOPS project: general EORTC QLQ-C30 and EQ5D-5L, pancreas-specific EORTC QLQ-PAN26, HADS, and WOPS.
- Compliance to recurrence-focused surveillance, measured as the percentage of patients that either accepts or refuses participation in the intervention-arm, i.e., is willing to undergo recurrence-focused follow-up.
- Disease-free survival, defined as the interval between the date of PDAC resection and the date of first radiological signs of recurrence, or last follow-up if recurrence is not observed. Preferably, recurrence is proven by a biopsy showing pathological confirmation of recurrent PDAC. However, recurrence can be clinically confirmed with growth of a suspicious lesion on consecutive CT scans and elevation of CA 19–9 (> 37 U/ml) and/or activity on PET-CT following consensus in a multidisciplinary meeting.
- Clinical and radiological patterns of disease recurrence:
  - ◦ Asymptomatic recurrence is defined as PDAC recurrence detected by CT imaging during follow-up, in the absence of suspected symptoms. If PDAC recurrence is discovered due to a significant patient-initiated complaint that is new or has been increased in severity or frequency, disease recurrence is defined as symptomatic.
  - ◦ Localization of recurrence is categorized using five categories: (1) isolated locoregional recurrence: recurrence in the pancreatic remnant, locoregional lymph nodes, or surgical bed, such as soft tissue along the celiac or superior mesenteric artery, aorta, or around the pancreatojejunostomy site; (2) isolated distant recurrence: recurrence restricted to a single organ or site; (3) local + distant recurrence: simultaneous occurrence of both isolated locoregional recurrence and isolated distant recurrence; (4) multiple recurrence: recurrence at multiple distant sites; and (5) peritoneal carcinomatosis.
- The yield of CT surveillance and role of serum tumor marker testing in detecting PDAC recurrence, by calculating diagnostic accuracy values, i.e., sensitivity, specificity, positive predictive values, and negative predictive values.
- Eligibility for additional (experimental) treatment at time of recurrence diagnosis, based on the Eastern Cooperative Oncology Group (ECOG) or Karnofsky performance state, or inclusion criteria for study-related treatment of recurrence.
- Reasons to refrain from treatment for recurrence (i.e., non-eligibility, deteriorated condition, patient’s wish, doctors’ advice, progression during treatment, etc.).
- Patients’ tolerance to treatment for recurrence, measured by the reasons to stop treatment for recurrence (i.e., per protocol, deteriorated condition, patient’s wish, doctors’ advice, progression during treatment etc.).
- Morbidity associated with recurrence-focused treatment.
- Overall costs of recurrence-focused surveillance vs. costs as incurred with current non-standardized follow-up. Cost-effectiveness is examined through the EQ5D questionnaire and calculated using a Markov model.

## Participant timeline {13}

**Table:** 

	**STUDY PERIOD**									
	**Enrolment**	**Allocation**	**Post-allocation**							
**TIMEPOINT**	**Preoperative**	**Postoperative**	**3 months**	**6 months**	**9 months**	**12 months**	**15 months**	**18 months**	**21 months**	**24 months**
PACAP or PACOPS registry, TwiCs & PROMs informed consent	X									
Clinical evaluation	X		X	X	X	X	X	X	X	X
Histological evaluation (confirmation PDAC and R0-R1 resection)		X								
Informed consent for study-related surveillance (investigational arm)		X								
Labs: CA 19–9, extra blood sample	X		X	X	X	X	X	X	X	X
CT chest and abdomen	X		X	X	X	X	X	X	X	X
QoL questionnaires^a^	X		X	X	X	X		X		X

^a^The following questionnaires are used: non-disease specific HRQL (EQ-5D-5L), cancer-specific HRQL (EORTC QLQ-C30), tumor-specific HRQL (EORTC QLQ-PAN26), hospital anxiety and depression scale (HADS), worry of progression of cancer scale (WOPS; modified Dutch seven-item version of the six-item english cancer worry scale (CWS))

### Sample size {14}

**Fig. 2:**
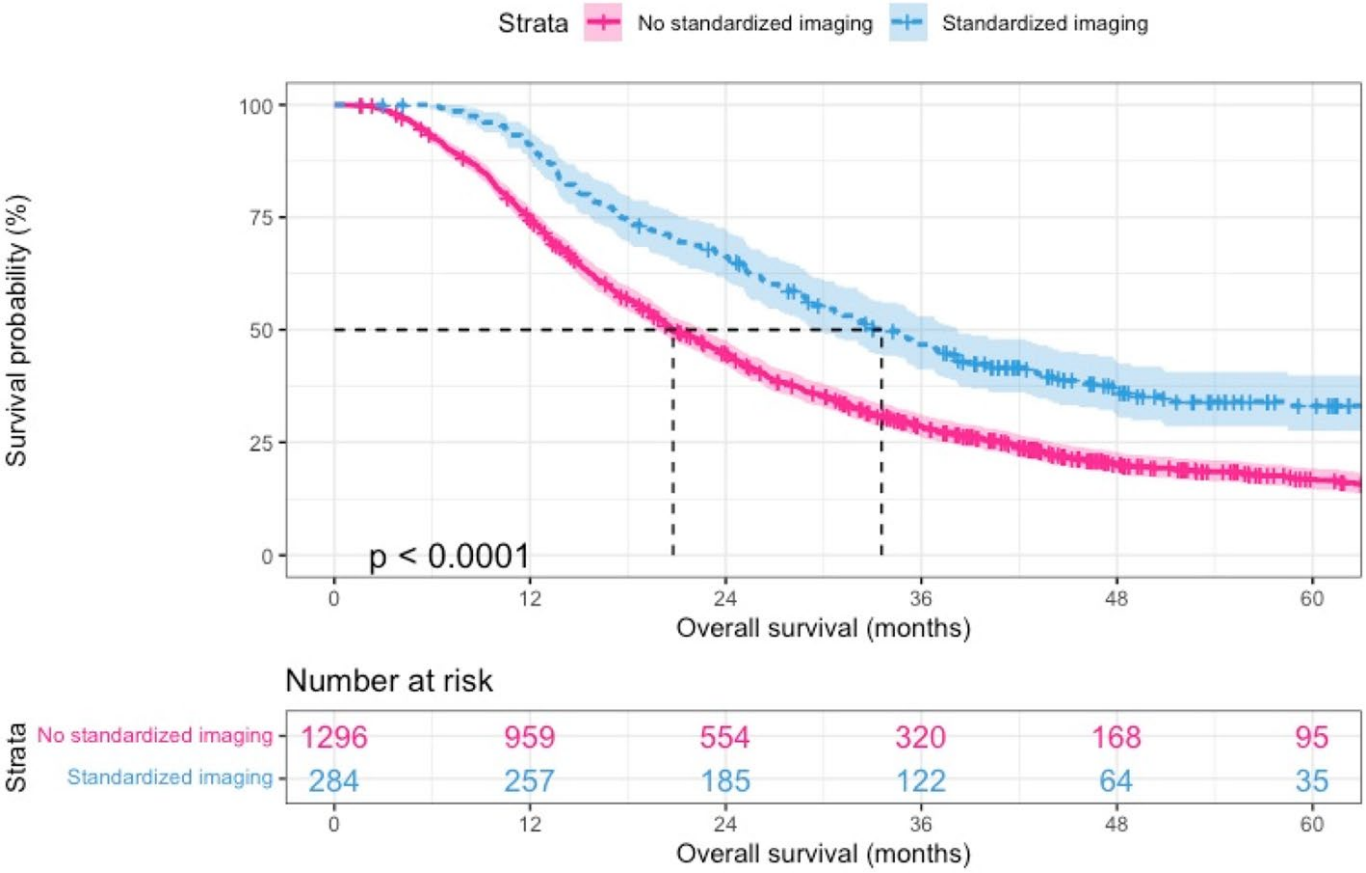
Kaplan–Meier curve comparing overall survival between patients with routine follow-up imaging and symptomatic follow-up in the Netherlands between 2014–2019 (unpublished data)

A retrospective study by Elmi et al. demonstrated that the median overall survival after primary tumor resection was 30.4 months in 163 patients who received routine imaging surveillance, as compared with 17.1 months in 66 patients receiving clinical follow-up without imaging [32]. Data from 1580 patients who underwent PDAC resection between 2014 and 2019 within the Netherlands showed a median overall survival of 33.6 months (95% confidence interval (CI) 29.4–38.2 months) in 284 patients who received routine follow-up imaging and 20.8 months (95% CI 19.8–22.6 months) in 1296 patients undergoing a symptomatic follow-up approach without routine imaging (Fig. 2). Combining these outcomes results in an expected median survival of 32.0 months vs. 19.0 months for patients in the intervention and control arm of the RADAR-PANC trial, respectively.

As we expect 80% of patients to accept the experimental intervention offered in the intervention arm of the study, an estimated refusal rate of 20% needs to be taken into account. This leads to a decrease of the overall survival to 29.4 months (80% × 32.0 months + 20% × 19.0 months) for patients in the intervention arm. Moreover, despite that current non-standardized follow-up in daily clinical practice mainly results in a symptomatic follow-up approach without routine imaging, data from our recurrence database showed that 85/1580 patients (5%) did nonetheless receive routine follow-up imaging based on shared-decision making (the remaining 199/284 patients (70%) who received routine follow-up imaging participated in a clinical study with a study-specific follow-up including routine imaging). This results in a slight increase of the suspected overall survival in the control arm to 19.7 months (95% × 19.0 months + 5% × 32.0 months). The clinically relevant survival difference of 29.4 months vs. 19.7 months for the intervention and control arm, respectively, also approximates the mean survival difference of 9.5 months presented in the meta-analysis from Halle-Smith et al. [33]. These numbers correspond to a relative hazard (RH) of survival of 1.49, which is used to calculate the sample size of the study. Furthermore, as we expect a minimal level of loss to follow-up in this surveillance study, a censoring rate of 1% is expected each month.

To detect a 49% overall survival improvement (RH of survival 1.49) for patients in the intervention group, as compared with the control group, with a statistical power of 80% and a 0.05 two-sided significance level, a sample size of 306 patients is required. This calculation was based on the assumption of an exponential model, a median overall survival of 19.7 months in the control arm, a follow-up duration of 30 months, a drop-out rate of 1% and a baseline event rate of 3.5%. Following this calculation, we plan to include 306 patients in total: 153 patients in the recurrence-focused follow-up group and 153 patients in the non-standardized, symptomatic follow-up group.

### Recruitment {15}

To achieve adequate patient enrolment and reach the target sample size, all centers are encouraged to participate in the current trial. To ensure enrolment of all eligible patients, the study coordinator(s) are in close contact with the local investigators at the participating centers to obtain weekly updates about patients who underwent macroscopically radical PDAC resection. Eligible patients will be contacted by the study coordinators in consultation with the treating clinicians. If an eligible patient is not yet approached for participation in the PACAP or PACOPS project, informed consent for PACAP or PACOPS, including TwiCs and PROMs, will be discussed by the study coordinator(s).

In 2018, 2348 patients were diagnosed with pancreatic cancer in the Netherlands. About 20% (*n* = 470) of these patients underwent macroscopically radical (R0-R1) resection of the tumor. In the Netherlands, all pancreatic resections are performed in centers affiliated with the DPCG, which will all participate in the current study. Based on the current successful enrolment progress, we anticipate that 75% (*n* = 353) of all patients will be registered yearly in the PACAP cohort and that 80% (*n* = 282) of these patients will provide informed consent for the TwiCs design. About 150 patients each year are expected to be included in other studies with a study-specific follow-up, which is an exclusion criterium for the RADAR-PANC trial. This leaves 282 – 150 = 132 patients eligible for randomization in the current study each year. Fifty percent (*n* = 66) of patients will be randomized to a standardized surveillance strategy. We expect that 80% (*n* = 53) of these patients are willing to participate in the intervention arm of the study, with a participation rate of 100% (*n* = 66) in the control arm as a consequence of the TwiCs design. The expected time needed for inclusion of a total of 306 patients (153 patients in each arm) is therefore 28 months. The final analysis will be performed 30 months after the last patient is enrolled.

### Assignment of interventions: allocation

#### Sequence generation {16a}

The allocation sequence to randomly assign participants into groups is based on computer-generated random numbers, following 2–4-6 variable block randomization, with stratification for institute. Eligible patients are randomized in chronological order.

### Concealment mechanism {16b}

According to the TwiCs design, only participants randomized to the experimental group will be informed explicitly. Patients randomized for the recurrence-focused follow-up will be notified and asked for additional informed consent. In contrast, patients randomized to the symptomatic surveillance (i.e., current clinical practice) will not be informed about their randomization specifically since they have already provided their consent to be randomized and collect their data in a standardized fashion. Consequently, there is no need to conceal participants’ allocation.

### Implementation {16c}

The allocation sequence is computer-generated. The central research coordinator enrolls eligible patients into the study and assigns participants to interventions. Patients who are randomly allocated to the intervention arm are offered the intervention by the research coordinator and asked to provide additional informed consent for participation in the intervention arm of the trial.

## Assignment of interventions: blinding

### Who will be blinded {17a}

Blinding is not applicable to this TwiCs study. As part of the TwiCs design, however, patients randomized to the control arm and their treating clinicians will not be further notified.

### Procedure for unblinding if needed {17b}

Unblinding is not applicable to this TwiCs study as blinding is not applied.

### Data collection and management

#### Plans for assessment and collection of outcomes {18a}

Baseline characteristics of all trial participants are standardly collected as part of the PACAP and PACOPS projects. Also, quality of life is already assessed using PROMs at standardized time points in all participants. Data management is carried out in accordance with UMC Utrecht Data management policy, as described in the Data Management Plan. Data is collected using a predefined, electronic case record form in Castor EDC. Local clinicians in the participating centers are responsible for data collection. They can, however, transfer this responsibility to the study team. The study team will appoint appropriate personnel for data collection.

### Plans to promote participant retention and complete follow-up {18b}

The central research coordinator plays an essential role in participant retention and complete follow-up. All follow-up appointments will be scheduled by the research coordinator in consultation with the treating clinician and patient. Participants of the study are therefore followed closely by the research coordinator. In case test results are available, the research coordinator ensures that the results are discussed with the patient and in a multidisciplinary team meeting, if desired.

### Data management {19}

Data management will be carried out in accordance with the UMC Utrecht Data management policy, as described in the Data Management Plan. Data will be collected using a predefined, electronic case record form in Castor EDC. Subjects will be entered into the patient database at each of the participating centers. Local personnel per participating center is responsible for data collection. They can, however, delegate this to the study steering committee who will appoint an appropriate data management team per site. All data will be collected using a predefined, electronic case record from. Data will be handled confidentially and coded. The data will be kept for a minimum of 15 years.

### Confidentiality {27}

The handling of personal data will comply with the Regulation (EU) 2016/679 of the European Parliament and of the Council of 27 April 2016 on the protection of natural persons with regard to the processing of personal data and on the free movement of such data (General Data Protection Regulation). A subject identification code list will be used to link the data to the subject. These codes will not be based on the patient initials and birth date. The local investigators will safeguard the key to this code.

### Plans for collection, laboratory evaluation, and storage of biological specimens for genetic or molecular analysis in this trial/future use {33}

Not applicable.

### Statistical methods

#### Statistical methods for primary and secondary outcomes {20a}

The latest version of R Studio will be used for statistical analysis.

**Fig. 3:**
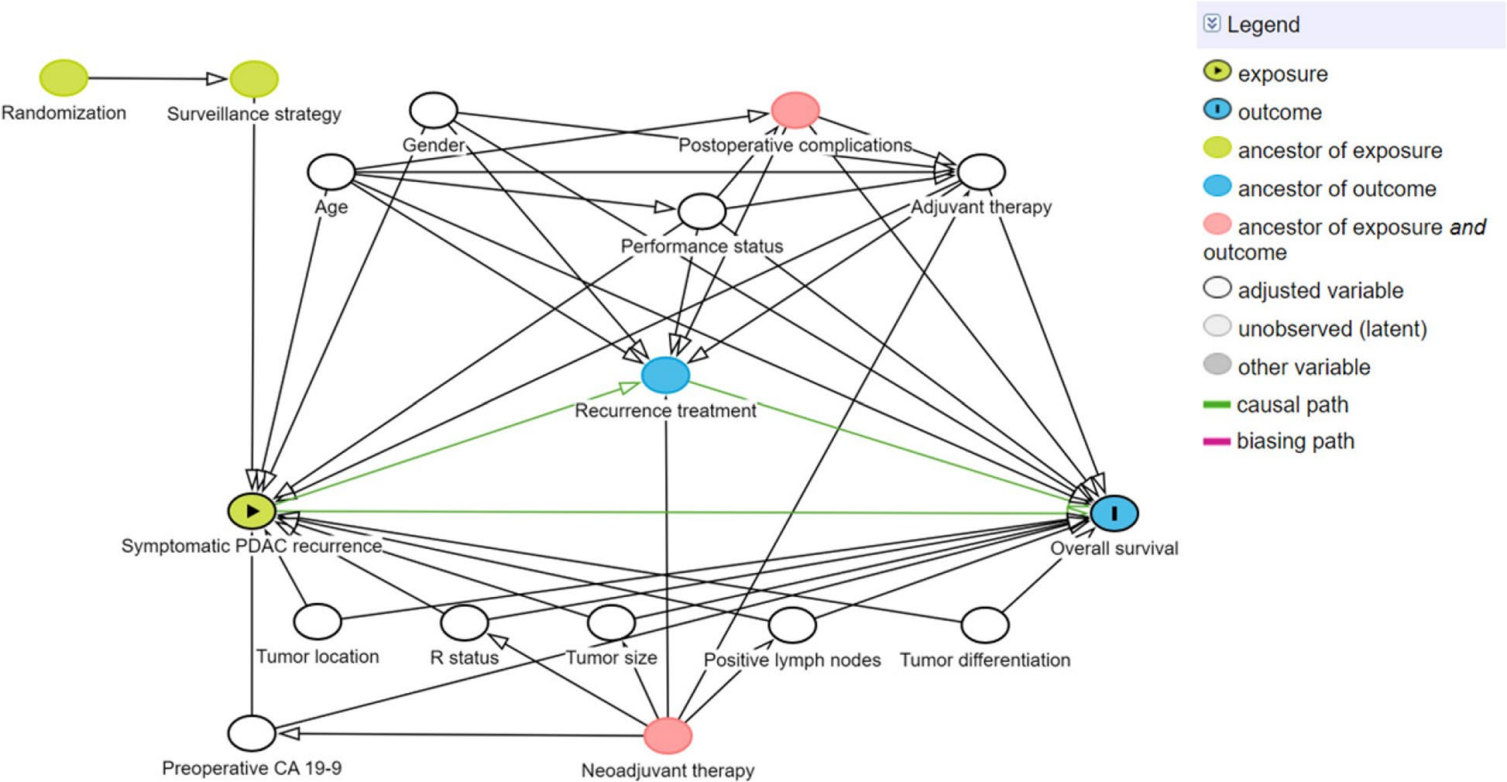
Directed acyclic graph to address potential confounders/effect modifiers to adjust in the multivariable analysis

Baseline characteristics will be reported using standard descriptive statistics. Success of randomization will be evaluated by eye-balling whether the distribution of baseline characteristics between the intervention and control group is even. Primary analysis will be performed according to the intention-to-treat principle. As such, patients randomized to the intervention arm of the trial who refuse recurrence-focused surveillance following the TwiCs design will be analyzed as part of the intervention group. The main study parameter is overall survival, measured as the time from the date of PDAC resection to the date of death. Patients are censored at the time of last known follow-up visit documented if no event occurred. Overall survival rates will be reported as median with 95% CI and will be calculated by the Kaplan–Meier survival curve method. Log-rank test will be used to compare survival between the intervention and control group. One- and 2-year survival probabilities will be calculated. Univariate Cox-proportional hazard analysis will be used to assess the crude effect of a recurrence-focused surveillance strategy on survival. In addition, adjusted effect estimates will be calculated using a multivariable Cox-proportional hazard model, including potential confounders for symptomatic PDAC recurrence. To address potential confounders for which the multivariable analysis needs to be adjusted, a Directed Acyclic Graph was designed on https://www.dagitty.net (Fig. 3). It is hypothesized that a recurrence-focused postoperative surveillance strategy leads to the detection of PDAC recurrence in an asymptomatic stage, which increases the probability to undergo treatment for recurrence, possibly improving survival. The association between postoperative surveillance strategy and overall survival is therefore fully mediated through presence of symptoms at the time of recurrence diagnosis and subsequent administration of recurrence treatment. Given that the median overall survival after resection of PDAC is 21 months and 1- and 2-year survival rates are 75% and < 50%, respectively, we expect that during our follow-up period at least 50% of included patients (*n* = 153) will experience an event. The number of variables included in the multivariable analysis is based on the rule of thumb of ~ 10 events per variable, where a categorical variable counts for the number of categories. Consequently, multivariable analysis is adjusted for 15 baseline covariates that are considered to be the most important, i.e., age (continuous), sex (male/female), and predictors that reflect an (un)favorable tumor biology or treatment course, including performance status (continuous), preoperative CA 19–9 value (continuous), tumor location (head/distal), resection margin status (R0/R1), tumor size (continuous), number of positive lymph nodes (continuous) and tumor differentiation (poor/well-moderate), and administration of adjuvant therapy (yes/no). Given that recurrence treatment is considered a mediator, and not a confounder, no adjustment for recurrence treatment is required. Because administration of neoadjuvant therapy is known to affect preoperative CA 19–9 values, resection margin status, tumor size, and number of positive lymph nodes, neoadjuvant treatment will not be adjusted for. Postoperative complications are deemed to affect the receipt of adjuvant therapy and will thus not be included in multivariable analysis. Results will be presented as hazard ratio’s (HR) with corresponding 95% confidence intervals (CI). A two-tailed *P-*value < 0.05 will be considered statistically significant.

Data on the incidence of PDAC recurrence, compliance to a recurrence-focused surveillance strategy, clinical, and radiological patterns of recurrence, eligibility for treatment of recurrence, administration of recurrence treatment, as well as type of treatment, reasons to refrain from recurrence treatment, and patient tolerance to recurrence treatment (i.e., completion of treatment as planned) will be analyzed and reported using descriptive statistics. Chi-square or Fisher’s exact test are used to compare categorical variables as appropriate. Parametric continuous variables are presented as mean ± standard deviation (SD) and are compared using the Student’s *t*-test. Non-parametric continuous variables are presented as median with interquartile range (IQR) and are compared using the Mann–Whitney *U* test. Patient-reported outcomes at each time point during follow-up will be compared to baseline scores using a generalized linear mixed model approach to assess a difference in patients’ quality of life and worry of cancer recurrence between the trial arms. Scores will be presented as mean (± SD), changes from baseline as mean differences (MD) and estimates will be calculated to represent a difference in MD between groups with corresponding 95% confidence intervals (CI). Minimally important differences (MIDs) for the EORTC QLQ-C30 will be interpreted based on Musoro et al. for available quality of life domains [34]. If not specified, a ≥ 10-point change is considered meaningful. Disease-free survival and post-recurrence survival will be assessed using Kaplan–Meier survival curves and reported as median with 95% CI. The log-rank test will be used to compare groups. Cox proportional hazards model will be used when appropriate. Stratified analyses will be performed regarding pattern of disease recurrence and type of additional treatment. Various baseline parameters (e.g., sex, age, body mass index (BMI), Charlson Comorbidity Index (CCI), ECOG score, postoperative complications, (neo)adjuvant therapy, resectability status, preoperative CA 19–9 values, tumor location, resection margin status, vascular resection, tumor (T) stage, lymph node (N) stage, tumor differentiation, perineural growth) will be collected. The yield of CT surveillance and role of serum tumor marker testing is assessed by calculating diagnostic accuracy values, i.e., sensitivity, specificity, false-positive and false-negative values. Cost-effectiveness is calculated using a Markov model.

### Interim analyses {21b}

As the primary endpoint is overall survival, reasonable follow-up time is necessary to evaluate this endpoint. When reaching 50% of inclusions, an interim analysis would not provide any information on the efficacy or futility of a recurrence-focused surveillance strategy since the corresponding information on overall survival of the patients included so far is still very limited. Therefore, no interim analysis was conducted.

### Methods for additional analyses (e.g., subgroup analyses) {20b}

The association between recurrence-focused postoperative surveillance, with subsequent detection of (a)symptomatic PDAC recurrence, and overall survival is anticipated to be at least partly mediated by the administration of treatment for recurrence (Fig. 3). To confirm this, we will perform a sub-analysis in which we stratify for recurrence treatment. As PDAC is known to be a highly heterogeneous disease, the underlying tumor biology might also influence the occurrence of (a)symptomatic disease recurrence and subsequently impact survival. To determine which part of the effect is mediated through recurrence-focused treatment (indirect effect) and which part can be attributed to tumor biology (direct effect), a causal mediation analysis will be performed to decompose the total effect. This will provide us further information on the true value of additional treatment for recurrence.

Moreover, the time interval between diagnosis of recurrence and start of treatment for recurrence (directly after detection in case of asymptomatic disease recurrence or as soon as symptoms occur) might affect the outcome. We therefore will perform a sensitivity analysis in which we include this time interval as a time-varying covariate.

### Methods in analysis to handle protocol non-adherence and any statistical methods to handle missing data {20c}

Analyses will be performed according to the intention-to-treat principle. Patients who refuse the offered intervention (i.e., a recurrence-focused surveillance according to our study protocol) will therefore be analyzed as participating in the intervention-arm of the trial, although they receive a non-standardized, symptomatic follow-up. Moreover, despite that non-standardized surveillance in general results in a symptomatic follow-up strategy without routine follow-up imaging in daily clinical practice, a proportion of patients in the control arm will receive recurrence-focused surveillance based on shared decision-making. Since patient crossover might lead to effect dilution, our sample size is corrected for refusal in the intervention arm and agreement on routine imaging in the control arm. Besides the intention-to-treat analysis, a per protocol analysis will be performed of only those who strictly adhered to the protocol to estimate the true efficacy of the recurrence-focused follow-up strategy. Missing baseline data will be imputed using multiple imputation techniques. Both complete data analysis and multiple imputed data analysis will be performed to check for inconsistencies.

### Plans to give access to the full protocol, participant level-data and statistical code {31c}

The full protocol, participant-level dataset, and statistical code can be requested from the principal investigator.

### Oversight and monitoring

#### Composition of the coordinating center and trial steering committee {5d}

The composition of the trial steering committee at the coordinating center exists of two PhD students from the coordinating center who are responsible for the day-to-day logistics of the trial, including identification of eligible patients, patient inclusion and randomization, and arranging follow-up appointments for patients randomized for the intervention arm. Additionally, they are supervised by an assistant professor and the principal investigator, with whom they meet on a weekly basis and can be consulted for discussion. The coordinating center is responsible for the correct coordination of the trial and providing oversight over all participating centers. The steering committee is assisted by clinicians from the coordinating center (i.e., the Regional Academic Cancer center Utrecht; RAKU), who are involved in the care of pancreatic cancer patients and are present at multidisciplinary team meetings, including surgeons, medical oncologists, radiation oncologists, radiologists, gastroenterologists, and pathologists, are easily accessible for consultation and support.

At each participating center, the local investigator is responsible for the on-site logistics and will appoint an appropriate trial team, which can in part be delegated to the coordinating center. Members of the DPCG will be updated on the trial progress at least during the four yearly DPCG meetings, but can be contacted for consultation, if needed. Radiologists from the participating expert pancreatic cancer center will revise all follow-up CT scans of patients who underwent surgery in their center, if performed elsewhere. In case recurrence is detected, this will be discussed at a multidisciplinary team meeting, functioning as an expert panel for determining study endpoints. Data managers from the coordinating center are responsible for data management, together with the daily trial coordinators.

### Composition of the data monitoring committee, its role, and reporting structure {21a}

The independent Data Safety Monitoring Board (DSMB) will consist of Prof. Dr. M.J.C. Eijkemans, epidemiologist/statistician, Dr. P.J. Blankestijn, Dr. J.F. Swart, and Dr. N. Haj Mohammad. The DSMB will meet once yearly and will review the progress and accruing data of this trial and provide advice on the conduct of the trial to the Trial Steering Committee. The role of the DMSB will be to perform an interim review of the trial’s progress including updated figures on recruitment, data quality, and main outcomes and safety data.

### Adverse event reporting and harms {22}

Adverse events in the intervention arm are assessed and recorded by the local investigator and, if necessary, discussed with a radiologist or surgeon. The following aspects will be recorded for each event:

- description and grade of the toxicity according to the Common Terminology Criteria for Adverse Events (CTCAE) version 5.0 [35];
- date of onset;
- date of recovery;
- action taken (intervention);
- outcome of the adverse event.

The sponsor will report serious adverse events in the intervention arm through the web portal *ToetsingOnline* to the accredited medical research ethics committee (MREC) (in Dutch: medisch ethische toetsing commissie, METC) that approved the protocol, within 7 days of first knowledge for serious adverse events that result in death or are life threatening followed by a period of maximum of 8 days to complete the initial preliminary report. All other serious adverse events will be reported within a period of maximum 15 days after the sponsor has first knowledge of the serious adverse event. As PDAC patients have a very poor prognosis, we expect that many patients suffer from follow-up unrelated SAEs within the 2-year study period. These SAEs will be recorded, although not reported. Adverse events will be followed until they have abated or until a stable situation has been reached. Depending on the event, follow-up may require additional tests or medical procedures as indicated and/or referral to the general physician or a medical specialist. Serious adverse events need to be reported until the end of the study within the Netherlands, as defined in the protocol. As the control arm of the trial is treated according to current standard of care, adverse events in the control group will not be reported.

### Frequency and plans for auditing trial conduct {23}

To assure quality and validity of research data, an independent, qualified monitor is appointed to monitor the study procedures. Monitoring is performed according to the Nederlandse Federatie van Universitaire Medische Centra (NFU) guidelines. During monitoring, the inclusion rate, study files, informed consent, in- and exclusion criteria, data review and data verification, serious adverse events, research procedures, research data, and research equipment will be checked for a random sample of patients. The risk of the study is classified as “negligible,” and minimal monitoring is therefore needed. Minimal monitoring exists of an initiation visit for each participating center, at least two on-site visits in the coordinating center (depending on the trial duration, the number of study participants and observed deviations), and a closing visit. At any given point during the study, the trial can be selected for audit. There is no predefined schedule for audits and inspections.

### Plans for communicating important protocol amendments to relevant parties (e.g., trial participants, ethical committees) {25}

Amendments are changes made to the research after a favorable opinion by the accredited MREC has been given. All amendments will be notified to the MREC that gave a favorable opinion.

### Dissemination plans {31a}

To disseminate the knowledge obtained, we will report the results from this project in one or more manuscripts, which will be submitted to high-impact, peer-reviewed journals. Results will also be discussed at regular meetings of the DPCG, and other national and international meetings, and will be submitted for presentation to (inter)national conferences. Also, the results may be included in treatment guidelines for PDAC.

## Discussion

The RADAR-PANC trial investigates whether a recurrence-focused surveillance strategy with serial tumor marker testing and routine imaging improves overall survival in patients after radical (R0/R1) resection of PDAC in the Netherlands, compared to current, non-standardized follow-up. Furthermore, the consequences of a recurrence-focused surveillance strategy on quality of life and recurrence-focused (experimental) treatment are assessed.

The impact of recurrence-focused surveillance has been prospectively investigated in patients who received treatment for other cancer types, including colorectal cancer and epithelial ovarian cancer. These studies did not demonstrate a disease-specific survival benefit of multimodality surveillance with a certain interval and cost-effectiveness of a recurrence-focused surveillance strategy remained unclear [36–39]. Unfortunately, the risk of disease recurrence after PDAC resection is substantially higher than for other cancer types. If timely treatment of disease recurrence in PDAC patients leads to improved survival, recurrence-focused follow-up might be more beneficial for these patients. Given that prospective studies on this subject are lacking, conclusions about the potential advantages of recurrence-focused surveillance after PDAC resection cannot be made. The RADAR-PANC trial will be the first randomized controlled trial to generate high level evidence for the current clinical equipoise regarding the value of recurrence-focused postoperative surveillance with serial tumor marker testing and routine imaging in patients after PDAC resection.

During the initiation of this study, several issues have been debated. First of all, the main study endpoint has been an important subject of discussion. Clearly, recurrence-focused surveillance alone does not lead to improved survival. Early recurrence detection following recurrence-focused surveillance, however, holds the potential to increase the eligibility for early initiation of recurrence-focused treatment, which might be associated with survival benefits. The efficacy and optimal timing of recurrence treatment has not been studied extensively and remains a subject of discussion. As a consequence, recommendations on treatment for recurrence are lacking in current PDAC guidelines [15]. Considering that the RADAR-PANC trial may lead to the detection of recurrence before the onset of symptoms in a substantial part of patients, discussions were held on how to treat patients with asymptomatic disease recurrence. The potential survival benefits of recurrence-focused postoperative surveillance are anticipated to result from early initiation of recurrence treatment. It was therefore decided that all patients in whom disease recurrence is diagnosed should be offered (experimental) treatment or participation in a clinical intervention trial. Within the Netherlands, the DPCG has simultaneously initiated two randomized controlled trials on respectively the optimal timing of systemic therapy for patients with disseminated disease, i.e., the TIMEPAN trial (NCT04897854), and the efficacy of SBRT for the treatment of isolated local PDAC recurrence in addition to standard of care, i.e., the ARCADE trial (NCT04881487). Nevertheless, while it is strongly encouraged to offer all trial participants treatment at time of recurrence diagnosis, type, and timing of treatment will be determined through shared decision-making. This might reduce the potential survival benefits of recurrence-focused surveillance, although insights into the impact on patients’ quality of life will be obtained.

In addition, it was discussed whether the main endpoint should be overall survival, defined as the interval between the date of resection and the date of death, or post-recurrence survival, defined from the date of recurrence diagnosis until date of death. The date of recurrence diagnosis, however, is highly dependent on the applied surveillance strategy and post-recurrence survival will therefore be subjected to lead time bias. Another outcome that was taken into consideration as potential main endpoint was quality of life. One of the most important reasons to refrain from recurrence-focused surveillance is the negative impact it might have on the quality of life of patients with an already poor prognosis. Nevertheless, a sample size can be calculated more reliably by using a hard endpoint such as survival instead of a soft outcome as quality of life. We therefore decided to make quality of life the most important secondary endpoint. In addition, this will be the first study to investigate the impact of a recurrence-focused surveillance strategy on the quality of life of patients after PDAC resection. As previous studies and relevant numbers on this subject are lacking, a proper sample size calculation with quality of life as main study endpoint could not be performed.

Lastly, the use of the TwiCs design has been extensively discussed. TwiCs is an innovative study design that has been proposed by Relton et al. in 2010 as a potential solution for certain logistical challenges in traditional randomized trials [23]. Theoretical advantages of TwiCs include efficient patient recruitment, considering that an observational patient cohort is used as multiple trials facility, which also leads to increased generalizability of results (less selection bias), and efficient use of routinely collected data [22]. The two-staged, patient-centered informed consent procedure is specifically chosen to prevent disappointment bias and contamination [25]. Especially in the setting of a trial on recurrence-focused postoperative surveillance, recruitment in a traditional RCT would be challenging, given that patients who have been informed about the potential benefits of surveillance may not be willing to accept randomization. In setting-up the RADAR-PANC trial, however, ethical concerns with regard to the TwiCs design were raised by some of the involved pancreatic cancer clinicians. It was questioned whether it was ethically acceptable that patients who are randomized to the control arm of the trial are not explicitly notified about their trial participation. At cohort enrolment, however, patients are well informed that they can be serving as control without further notifications. In addition, a survey among cancer patients who served as control in previous TwiCs showed that the majority of patients considered this to be acceptable [26]. Nevertheless, explanation to and education of researchers and physicians is of great importance to overcome ethical dilemmas.

### Trial status

Protocol version 4.0, October 24, 2023. The first patient was enrolled on March 16, 2021. At the time of submission of this manuscript (January 2, 2024), 219 patients have been enrolled by 7 institutions. Recruitment is anticipated to be completed in April 2024.

## Data Availability

The full protocol, final trial dataset, and statistical code will be accessible for investigators and can be obtained upon reasonable request from the principal investigator.

## Abbreviations

ASCO: American Society of Clinical Oncology
Asymptomatic PDAC recurrence: PDAC recurrence detected by CT imaging during follow-up, in the absence of suspected symptoms
BMI: Body mass index
BSC: Best supportive care
CA 19-9: Carbohydrate antigen 19–9
CCI: Charlson comorbidity index
CI: Confidence interval
Clinically confirmed recurrence: 2/3 Of the following criteria: -Growth of a suspicious lesion on consecutive CT scans, -Elevation of CA 19–9 (> 37 U/ml), -Abnormal activity on PET-CT
CT: Computed tomography
CTCAE: Common Terminology Criteria for Adverse Events
CWS: Cancer worry scale
Disease-free survival: The interval between the date of surgery and either the date of first radiological signs of recurrence, or last follow-up if recurrence is not observed
DPCG: Dutch Pancreatic Cancer Group
DSMB: Data Safety Monitoring Board
EORTC QLQ-C30: Cancer-specific health-related quality of life
EQ-5D-5L: Non-disease specific health-related quality of life
ECOG: Eastern Cooperative Oncology Group
ESMO: European Society for Medical Oncology
FOLFIRINOX: Fluorouracil, leucovorin, irinotecan, and oxaliplatin
HADS: Hospital anxiety and depression scale
HQRL: Health-related quality of life
HR: Hazard ratio
IAP/EPC: International Association of Pancreatology/European Pancreatic Club
IQR: Interquartile range
MD: Mean differences
MID: Minimally important difference
MREC/METC: Medical research ethics committee (MREC); in Dutch: medisch ethische toetsing commissie (METC)
MRI: Magnetic resonance imaging
NCCN: National Comprehensive Cancer Network
NFU: The Netherlands Federation of University Medical Centers; in Dutch: Nederlandse Federatie van Universitaire Medische Centra
NVvH: In Dutch: De Nederlandse Vereniging voor Heelkunde
Overall survival: The time from the date of resection of PDAC until either the date of death from any cause or date of last follow-up
PACAP: Dutch Pancreatic Cancer Project
PACOPS: Pancreas Cancer: Observations of Practice and Survival
PDAC: Pancreatic ductal adenocarcinoma
PDAC recurrence: Pathologically or clinically proven recurrence of PDAC
PET-CT: Positron emission tomography—computed tomography
PROMs: Patient reported outcome measurements
QoL: Quality of life
Radical resection: Macroscopically radical resection, i.e., R0-R1 resection
RAKU/RACU: Regional Academic Cancer center Utrecht
RH: Relative hazard
UK: United Kingdom
SBRT: Stereotactic Body Radiation Therapy
SD: Standard deviation
Sponsor: The sponsor is the party that commissions the organization or performance of the research, for example a pharmaceutical company, academic hospital, scientific organization or investigator. A party that provides funding for a study but does not commission it is not regarded as the sponsor, but referred to as a subsidizing party
Symptomatic recurrence: Recurrent PDAC discovered due to a significant patient-initiated complaint that is new or have been increased in severity or frequency
TwiCs: Trials within Cohorts
WMO: Medical Research Involving Human Subjects Act (in Dutch: Wet Medisch-wetenschappelijk Onderzoek met Mensen
WOPS: Worry of progression of cancer scale
