# Correction: Recurrent disease detection after resection of pancreatic ductal adenocarcinoma using a recurrence-focused surveillance strategy (RADAR-PANC): protocol of an international randomized controlled trial according to the Trials within Cohorts design

**DOI:** 10.1186/s13063-024-08295-3

**Published:** 2024-07-05

**Authors:** L. A. Daamen, I. W. J. M. van Goor, V. P. Groot, P. C. M. Andel, L. A. A. Brosens, O. R. Busch, G. A. Cirkel, N. Haj Mohammad, H. D. Heerkens, I. H. J. T. de Hingh, F. Hoogwater, H. W. M. van Laarhoven, M. Los, G. J. Meijer, V. E. de Meijer, R. Pande, K. J. Roberts, J. Stoker, M. W. J. Stommel, G. van Tienhoven, R. C. Verdonk, H. M. Verkooijen, F. J. Wessels, J. W. Wilmink, M. G. Besselink, H. C. van Santvoort, M. P. W. Intven, I. Q. Molenaar, for the Dutch Pancreatic Cancer Group

**Affiliations:** 1 Department of Surgery, Regional Academic Cancer Center Utrecht, UMC Utrecht Cancer Center & St. Antonius Hospital Nieuwegein, Nieuwegein, the Netherlands; 2 Division of Imaging, UMC Utrecht Cancer Center, Utrecht University, Utrecht, the Netherlands; 3 Department of Radiation Oncology, Regional Academic Cancer Center Utrecht, UMC Utrecht Cancer Center & St. Antonius Hospital Nieuwegein, Nieuwegein, the Netherlands; 4 Department of Pathology, Regional Academic Cancer Center Utrecht, UMC Utrecht Cancer Center & St. Antonius Hospital Nieuwegein, Nieuwegein, the Netherlands; 5 Department of Pathology, Radboud University Medical Center, Nijmegen, the Netherlands; 6 Amsterdam UMC, Department of Surgery, Location University of Amsterdam, Amsterdam, the Netherlands; 7 Cancer Center Amsterdam, Amsterdam, the Netherlands; 8 Department of Medical Oncology, Regional Academic Cancer Center Utrecht, UMC Utrecht Cancer Center, St. Antonius Hospital Nieuwegein & Meander Medical Center, Utrecht University, Utrecht, the Netherlands; 9 Department of Radiation Oncology, Radboud University Medical Center, Nijmegen, the Netherlands; 10 Department of Surgery, Catharina Hospital, Eindhoven, the Netherlands; 11 Department of Surgery, University of Groningen, University Medical Center Groningen, Groningen, the Netherlands; 12 Amsterdam UMC, Department of Medical Oncology, Location University of Amsterdam, Amsterdam, the Netherlands; 13 Department of Hepatopancreatobiliary Surgery and Liver Transplantation, Queen Elizabeth Hospital, University Hospitals Birmingham, Birmingham, UK; 14 Institute of Immunology and Immunotherapy, University of Birmingham, Birmingham, UK; 15 Amsterdam UMC, Department of Radiology, Location University of Amsterdam, Amsterdam, the Netherlands; 16 Department of Surgery, Radboud University Medical Center, Nijmegen, the Netherlands; 17 Amsterdam UMC, Department of Radiation Oncology, Location University of Amsterdam, Amsterdam, the Netherlands; 18 Department of Gastroenterology and Hepatology, Regional Academic Cancer Center Utrecht, UMC Utrecht Cancer Center & St. Antonius Hospital Nieuwegein, Nieuwegein, the Netherlands; 19 Department of Radiology, Regional Academic Cancer Center Utrecht, UMC Utrecht Cancer Center & St. Antonius Hospital Nieuwegein, Nieuwegein, the Netherlands

**Correction** **:** **Trials 25, 401 (2023)**

**https://doi.org/10.1186/s13063-024-08223-5**

Following the publication of the original article [1], we were notified that figures 2 and 3 have been swapped. The original article was corrected.

Furthermore, under item 20a, the phrase “To address potential confounders for which the multivariable analysis needs to be adjusted, a Directed Acyclic Graph was designed on https://www.dagitty.net (Fig. 2)” should read “To address potential confounders for which the multivariable analysis needs to be adjusted, a Directed Acyclic Graph was designed on https://www.dagitty.net (Fig. 3)”.

The original article was corrected.
